# Low prevalence of *Plasmodium falciparum pfhrp*2 and *pfhrp*3 gene deletions in archived samples collected before and after nationwide malaria-RDT rollout in Kenya

**DOI:** 10.3389/fepid.2026.1817539

**Published:** 2026-09-18

**Authors:** Martha N. Kivecu, Victor Mobegi, Cavin Mgawe, Brenda M. Kania, Gladys C. Chemwor, Raphael O. Okoth, Edwin W. Mwakio, George Obiero, Jennifer M. Mutisya, Jackline A. Juma, Agnes C. Cheruiyot, Benjamin H. Opot, Amanda L. Roth, Bernhards R. Ogutu, Dennis W. Juma, Lynette Isabella Ochola-Oyier, Ben Andagalu, Hoseah M. Akala

**Affiliations:** 1 Malaria Drug Resistance program, Kenya Medical Research Institute (KEMRI)/Walter Reed Army Institute of Research – Africa (WRAIR–A), Kisumu, Kenya; 2 Centre for Biotechnology and Bioinformatics (CEBIB), University of Nairobi, Nairobi, Kenya; 3 Department of Biochemistry, University of Nairobi, Nairobi, Kenya; 4 Biosciences Department, KEMRI Wellcome Trust Research Programme, Kilifi, Kenya; 5 Center for Clinical Research, Kenya Medical Research Institute (KEMRI), Nairobi, Kenya

**Keywords:** histidine-rich protein 2, malaria rapid diagnostic tests, pfhrp2/pfhrp3 gene deletions, pfhrp2/pfhrp3 genotyping, plasmodium falciparum

## Abstract

Rapid and accurate malaria diagnosis is essential for effective treatment and malaria control. In Kenya, most malaria testing relies on *P. falciparum* histidine-rich protein 2 (PfHRP2)-based rapid diagnostic tests (RDTs). However, deletions in the *pfhrp*2/*pfhrp*3 genes can lead to false-negative RDT results, which can sustain transmission and worsen clinical outcomes. This study assessed the prevalence and pattern of *pfhrp*2, *pfhrp*3 and their respective flanking genes in archived samples collected before and after the nationwide rollout of PfHRP2-based RDTs in Kenya in 2012. A total of 350 archived whole-blood samples were analysed, comprising 95 pre-RDT samples (collected before the nationwide rollout of RDTs in Kenya) from 2003 to 2005 and 255 post-RDT samples (collected after the nationwide rollout of RDTs in Kenya) from 2013 to 2017 from multiple malaria-endemic regions of Kenya. Samples were first screened by quantitative Real-Time PCR to identify those with adequate parasite density for genotyping. Nested PCR assays were then used to genotype *pfhrp*2, *pfhrp*3 and their flanking genes. Where available, PfHRP2-based RDT results were compared with molecular genotyping outcomes to investigate potential causes of false-negative RDT results. The overall prevalence of single *pfhrp*2 or *pfhrp*3 deletions was low, at 0.3% (1/317) and 1.3% (4/317), respectively. All pre-RDT samples carried intact *pfhrp*2 and *pfhrp*3 genes. Among post-RDT samples, deletions were rare 0.4% (1/224) for *pfhrp*2 gene only and 1.8% (4/224) for *pfhrp*3 gene only*.* No sample exhibited a triple deletion of *pfhrp*2 and both of its flanking genes or triple deletion of *pfhrp*3 and both of its flanking genes. The sensitivity of the PfHRP2-based RDT was 89.2% (174/195). Notably, 95.2% of false-negative RDT results occurred in samples with intact *pfhrp*2 and *pfhrp*3, suggesting that low parasitemia or other factors, rather than gene deletions, were the primary cause of diagnostic failure. Overall, *pfhrp*2 and *pfhrp*3 deletions remained rare in the samples collected up to five years after the nationwide rollout of PfHRP2-RDTs in Kenya. However, continuous molecular surveillance is essential to identify emerging deletion hotspots and to ensure the sustained reliability of PfHRP2-based diagnostics as testing practices and parasites continue to evolve.

## Introduction

Effective case management is a cornerstone of malaria control and accurate diagnosis is therefore essential for guiding timely treatment and supporting community-level surveillance. Although microscopy remains the gold standard for malaria diagnosis, the WHO also recommends malaria Rapid Diagnostic Tests (RDTs) as a practical alternative in settings where microscopy is unavailable or impractical, particularly during large-scale community testing (1). Together, these diagnostic approaches have strengthened malaria diagnosis (2).

Malaria RDTs detect circulating parasite antigens such as *Plasmodium* genus-specific aldolase, *Plasmodium* lactate dehydrogenase (pLDH), and *P. falciparum* histidine-rich protein 2 (PfHRP2) (3). PfHRP2 is abundant and stable in peripheral blood, making it an excellent diagnostic target (4). More than 90% of RDTs used in Sub-Saharan Africa are PfHRP2*-*based, reflecting the predominance of *P. falciparum* in the region (2). The adoption of rapid diagnostic tests has increased the proportion of suspected malaria cases receiving malaria diagnosis from 36% in 2010 to 85% in 2018 (5, 6).

Although PfHRP2-based RDTs may detect PfHRP3 antigens due to epitope similarity, PfHRP3 expression is generally low, and is often not detectable at low parasite densities (7, 8). A growing challenge is the emergence of parasites with *pfhrp*2 and/ or *pfhrp*3 gene deletions that evade detection, resulting in false-negative RDT outcomes. First reported in Peru (3, 9), *pfhrp*2 and *pfhrp*3 deletions have since been detected across Sub-Saharan Africa, Southeast Asia, and South America. In Eastern Africa, Eritrea, Ethiopia, and Djibouti have reported high deletion prevalences, prompting national policy shifts away from PfHRP2-based RDTs (10–14)*.* Kenya, however, has previously reported deletion prevalence below the WHO 5% threshold (15–17). Nonetheless, Kenya is classified as a high-risk country for the potential expansion of parasites carrying these deletions (18). This study assessed *pfhrp*2 and *pfhrp*3 deletions in archived samples collected before and after the nationwide rollout of PfHRP2-based RDTs in Kenya in 2012, to provide insights into temporal patterns and potential diagnostic implications.

## Methodology

### Ethics approval

This study was conducted under the approval of the Kenya Medical Research Institute (KEMRI), Scientific and Ethics Review Unit (SERU) and Walter Reed Army Institute of Research (WRAIR) institutional review boards, protocol numbers: WRAIR 2454, KEMRI 3628: “Epidemiology of malaria and drug sensitivity patterns in Kenya.”

### Study sites and sample sources

The study was conducted at KEMRI, WRAIR-A, Kenya, Malaria Drug Resistance Laboratory in Kisumu. We analysed archived whole-blood samples from the approved malaria surveillance study (KEMRI SSC 1330, WRAIR 1384). These archived samples were collected from hospitals in Kisumu, Kombewa, Kericho, Malindi, and Alupe. Participants (≥6 months old) were eligible if they had tested positive for malaria by RDT (2013–2017) or microscopy (2003–2005) or were symptomatic but negative by initial testing. The study population represented high-transmission lake-endemic zones, epidemic-prone highlands, and coastal malaria regions (Figure 1).

**Figure 1:**
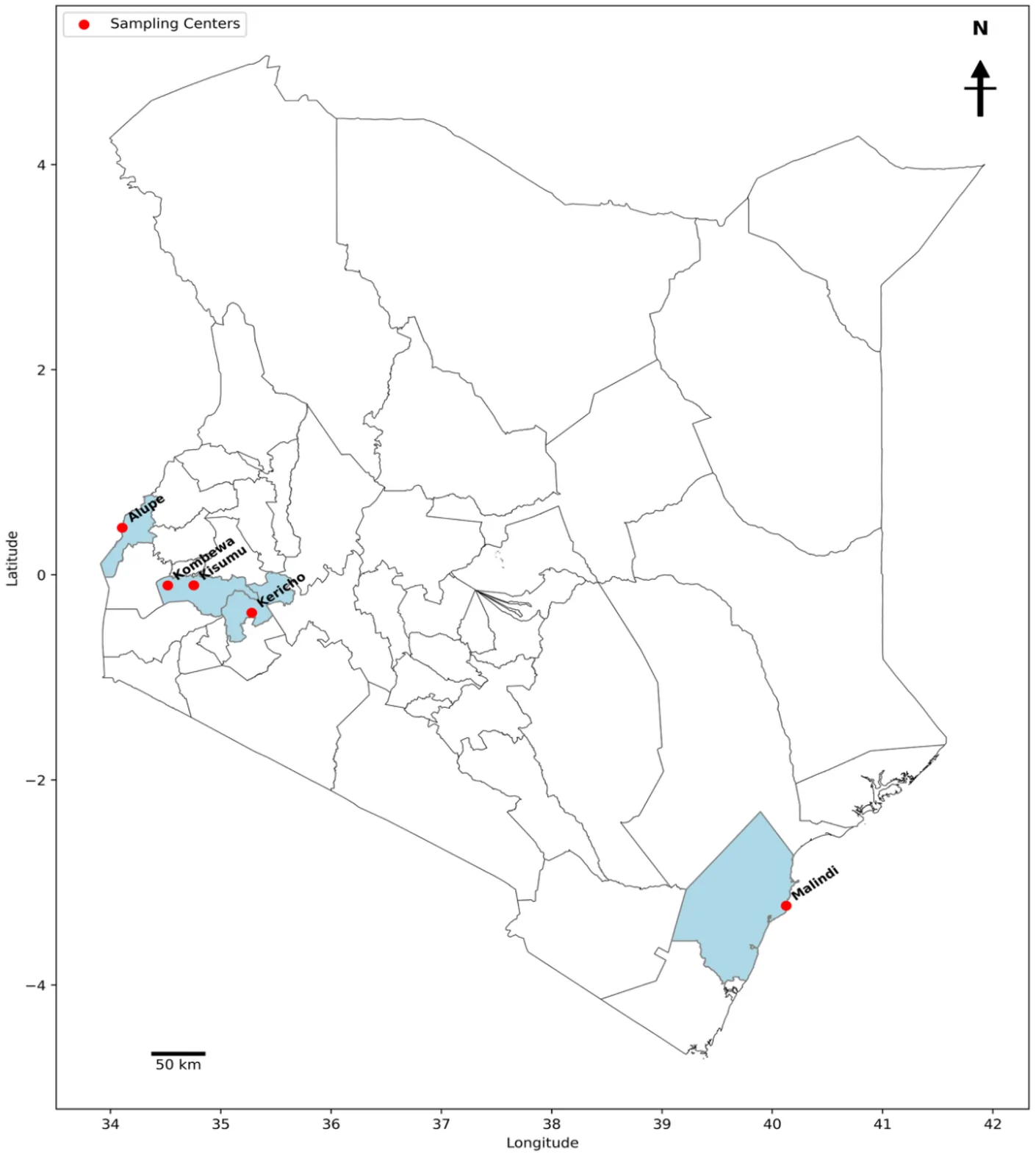
Geographic distribution of sampling sites across major malaria endemic zones in Kenya, including lake endemic (Alupe, Kombewa, and Kisumu), highland epidemic (Kericho), and coastal endemic (Malindi).

### Sample size and screening with Pan/PfHRP2 based RDT

A total of 350 samples were included: 95 DNA samples from 2003 to 2005 (pre-RDT era, prior to the nationwide rollout of RDTs in Kenya) and 255 whole blood samples from 2013 to 2017 (post-RDT era, after the nationwide rollout of RDTs in Kenya). Parascreen™ Pan/PfHRP2 Rapid Diagnostic Test (Zephyr Biomedicals, Goa India) were used to screen the whole blood according to the manufacturer's instructions. This RDT cassette detects two *P. falciparum* antigens: the first band detects the PfHRP2 antigen, the middle band detects the pan-*Plasmodium* lactate dehydrogenase enzyme (*P*LDH), and the third serves as the control line containing anti-rabbit antibody. Briefly, 5 µL of whole blood were added to the sample well of the RDT cassette, followed by two drops of buffer. The cassette was then incubated at room temperature for 20 min. Test results were interpreted based on the presence and pattern of bands, following the manufacturer's (Parascreen™) instructions.

### DNA extraction and quantitative real-time PCR (qPCR) screening

DNA was extracted using the Qiagen DNA Mini extraction spin protocol (Qiagen, Valencia, CA) according to the manufacturer's guidelines. Extracted DNA was eluted with 150 µL of elution buffer and stored at −20 °C until downstream PCR assays were conducted. *P. falciparum* infection was confirmed using 18S rRNA quantitative PCR (19, 20). The assay followed a two-step approach: an initial genus-specific amplification to detect the *Plasmodium* species, followed by a species-specific targeting *P. falciparum*. The real-time PCR assay was performed on an Applied Biosystem QuantStudio flex (QuantStudio™ real-time PCR applied Biosystems) under the following conditions: an initial denaturation at 95°C for 10 min, followed by 40 cycles of 95°C for 15 min, and then 60°C for 1 min. Each 10 µL reaction contained 0.4 µM of each primer and 0.2 µM of probe and 1 µL DNA template, and 4 µL of 2× QuantiFast master mix (Qiagen, catalogue no. 204257). The Cycle threshold (Ct) values were exported to Microsoft Excel for analysis, and samples with Ct ≤32 corresponding to approximately 30 parasites/µL (21) were considered adequate for *pfhrp*2/*pfhrp*3 genotyping.

### PCR amplification of *pfhrp*2, *pfhrp*3 and flanking genes

A nested PCR was conducted to confirm the presence or absence of *pfhrp*2, *pfhrp*3 and their flanking genes. The flanking genes are located upstream (5′) and downstream (3′) of *pfhrp*2 (specifically *Pf*3D7_0831900 and *Pf*3D7_0831700) and *pfhrp*3 (*Pf*3D7_1372400 and *Pf*3D7_1372100) as shown (Supplementary Figures S1, S2). The primers and cycling conditions used were based on a protocol described by Abdallah et al. (22), with two modifications for the *pfhrp*3 gene: (i) the annealing temperature in the primary amplification was increased to 60 °C, and (ii) the number of cycles increased from 30 to 35 to enhance amplification sensitivity for this locus. All PCR amplifications were performed in a total reaction volume of 20 µL. Each reaction contained QuantiFast master mix (Qiagen, catalogue no. 204257), 10 µM forward and reverse primers, and 1 µL of 34.0 ng/µL DNA template. The *P. falciparum* 3D7 strain was used as the positive control for all the assays. For *pfhrp*2 and its flanking regions, Dd2 (5 ng/µL), which has a known *pfhrp*2 deletion, was used as the negative control (23), and as a positive control for *pfhrp*3 and its flanking genes. Furthermore, the laboratory strain HB3 (5 ng/µL) which has the *pfhrp*3 deletion, was used as the negative control for *pfhrp*3 and a positive control for *pfhrp*2 and its flanking genes (24). Following PCR amplification, the products were visualised on 1.5% (w/v) agarose gel electrophoresis to determine the presence or absence of amplification for each target. The presence of a band at the expected product size was considered a positive result, indicating successful amplification (Supplementary Figures 3, 4). For samples that did not amplify on the first attempt, the PCR was repeated. If the second attempt was concordant with the first, that result was recorded as the final. If the first two results were discordant, a third assay was performed, and the outcome was determined by the two concordant results out of the three attempts.

### Data analysis

The prevalence of *pfhrp*2 and *pfhrp*3 gene deletions was calculated by dividing the number of samples with confirmed deletions in *pfhrp*2/*pfhrp*3 genes by the total number of samples that were positive for *P. falciparum* by 18S ribosomal RNA PCR. To assess the correlation between false-negative PfHRP2-based RDT results and *pfhrp*2/*pfhrp*3 deletion status, the following inclusion criteria were applied: 1) Samples testing positive for *P. falciparum* by 18S rRNA PCR with a parasite density of at least 30 parasites per microliter, a threshold selected because it corresponds to the approximate limit of detection for malaria RDTs; and 2) Samples testing positive for the *Plasmodium* genus by pan-based RDTs. Samples that met these inclusion criteria but produced a negative result on the PfHRP2-based RDT were classified as false negatives. Deletions of *pfhrp*2 and *pfhrp*3 were analysed using Microsoft Excel 2016. Firth-Corrected logistic regression was used to assess whether False-negative PfHRP2 based RDTs was associated with *pfhrp*2/*pfhrp*3 genes deletion using logistf R package (25, 26).

logistf(PfHRP2_RDT∼pfhrp2+pfhrp3,data=rdt_hrp2_3)

## Results

### Quantitative real-time PCR screening and sample inclusion

A total of 327 out of 350 samples (93.4%) were positive for *P. falciparum* by PCR (qPCR). This included 234 post-RDT samples collected between 2013 and 2017, and 93 pre-RDT samples collected in 2003–2005. The remaining 6.6% (23/350) of samples were negative for *P. falciparum* by qPCR and were excluded from further analysis (Figure 2). Of the 327 qPCR positive samples, 317 (96.9%) had a parasite count of at least 30 parasites per microliter and therefore met the criteria for analysis of RDT reactivity and *pfhrp*2 and *pfhrp*3 genotyping. The remaining 10 samples (3.1%) had less than 30 parasites per microlitre and were excluded from further analysis.

**Figure 2:**
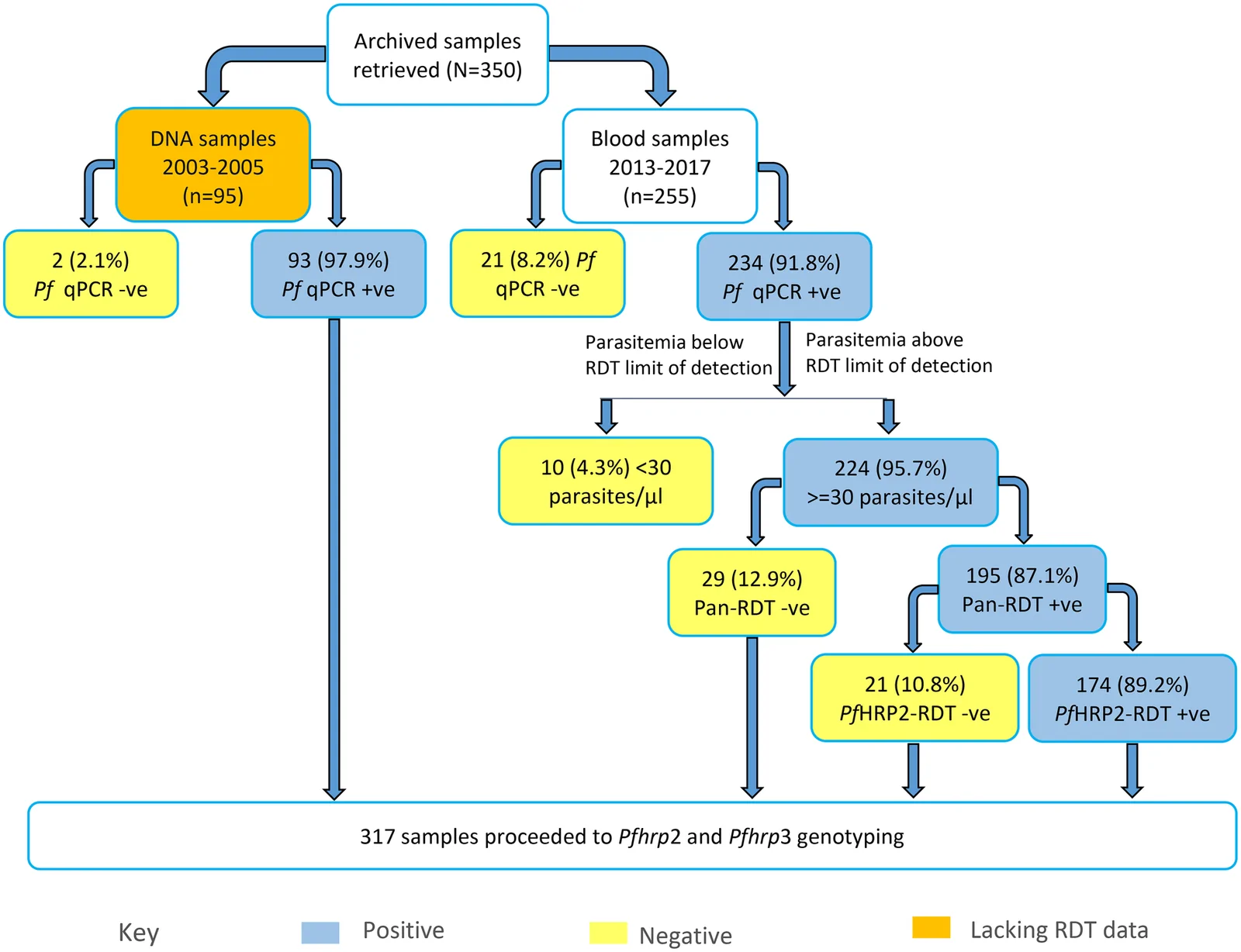
Flow diagram of *P. falciparum* qPCR and RDT results. A total of 350 (95 pre-RDT and 255 post-RDT) archived samples were screened for *P. falciparum* by qPCR. 224 post-RDT samples positive for *P. falciparum* qPCR with at least 30 parasites/µL were detected using Pan/*Pf*HRP2-based RDT. Out of 195 samples positive by Pan RDT, 21 (10.8%) samples were negative by PfHRP2-based RDT. RDT was not performed on the pre-RDT samples as only DNA was available.

### RDT results

Of the 350 samples analysed, 327 tested positive for *P. falciparum* by PCR. Among these, 317 (96.9%) met the parasite density threshold (Ct ≤32) for *pfhrp*2/*pfhrp*3 genotyping and RDT performance analysis. 224 (70.7%) samples were post-RDT samples whereas 93 (29.3%) were pre-RDT samples (Figure 2). RDT screening was not performed on 93 (29.3%) pre-RDT samples because only DNA was available. Among the 224 post-RDT samples, 195 (87.1%) were positive on the pan-LDH band, while 29 (12.9%) were negative. These 29 pan-negative samples were excluded from the analysis of PfHRP2-based RDT reactivity. Of the 195 pan-RDT and *P. falciparum* qPCR positive samples, 21 (10.8%) were negative on the PfHRP2-based RDT, indicating possible false-negative RDT results (Figure 2). The sensitivity of the PfHRP2-based RDT was 89.2% (174/195) when compared against pan-positive RDT results in *P. falciparum* PCR-positive samples Figure 2.

The overall prevalence of false-negative PfHRP2-based RDT results was highest in 2013, at 16.1% (10/62) and lowest in 2014, at 4.8% (2/42) (Table 1). No consistent temporal or spatial trend in PfHRP2-based RDT false negativity was observed across the period during which sampling was done. In Kericho, the prevalence decreased from 25.0% (5/20) in 2013 to 6.7% (1/15) in 2016. In contrast, the prevalence in Kombewa increased from 3.6% (1/28) in 2013 to 10% (3/30) in 2015, before decreasing slightly to 5.6% (1/18) in 2016. The Malindi site had no samples in 2014 and 2015, and only a few samples were recorded across all sites in 2017 (Table 1). Across all years, Malindi recorded the highest prevalence of false-negative RDT results at 31.6% (6/19), followed by Kericho at 13.7% (10/73) and Kombewa 4.9% (5/103) (Table 1).

**Table 1:** Spatial and temporal prevalence of the 21 false-negative PfHRP2-based RDT samples among the 195 Pan-RDT positive and *P. falciparum* qPCR positive samples.

Site	2013	2014	2015	2016	2017	Prevalence per study site
Malindi	4/14 (28.6%)	—	—	2/4 (50.0%)	0/1 (0.0%)	6/19 (31.6%)
Kombewa	1/28 (3.6%)	0/25 (0.0%)	3/30 (10.0%)	1/18 (5.6%)	0/2 (0.0%)	5/103 (4.9%)
Kericho	5/20 (25.0%)	2/17 (11.8%)	2/20 (10.0%)	1/15 (6.7%)	0/1 (0.0%)	10/73 (13.7%)
Yearly prevalence	10/62 (16.1%)	2/42 (4.8%)	5/50 (10.0%)	4/37 (10.8%)	0/4 (0.0%)	21/195 (10.8%)

“—” Indicates no samples were collected for that study site in that year. Values are presented as the number of false-negative PfHRP2-based RDT samples/total samples for a specific study site in a given year (percentage). Percentages represent prevalence within each study site in a given year. The last row represents the overall prevalence of false-negative PfHRP2-based RDT samples for each year, whereas the last column represents the overall prevalence of false-negative PfHRP2-based RDT for each study site.

### *Pfhrp*2 and *pfhrp*3 genotyping PCR

A total of 317 *P. falciparum* qPCR-positive samples were genotyped to assess deletions in the *pfhrp*2 and *pfhrp*3 genes. Of these, 311 (98.1%) samples were positive for *pfhrp*2, one (0.3%) sample was negative *pfhrp*2 gene, and *pfhrp*2 genotyping data were missing for the remaining five (1.6%) samples. Of 317 samples tested for *pfhrp*3, 310 (97.8%) were positive, four (1.3%) were negative for *pfhrp*3 gene, and *pfhrp*3 genotyping data was missing for three (0.9%) samples.

In total, 306 of 317 samples were positive for both *pfhrp*2 and *pfhrp*3 genes. Four samples had single deletion of *pfhrp*3 gene, one sample had single deletion of *pfhrp*2 gene, and no sample had double deletion of both *pfhrp*2 and *pfhrp*3 genes. Of the remaining six samples, two had missing genotyping data for both *pfhrp*2 and *pfhrp*3; three had missing genotyping data for *pfhrp*2 only, and one had missing data for *pfhrp*3 only (Table 2).

**Table 2:** *Pfhrp*2 and *pfhrp*3 genotyping profile.

*pfhrp*2 genotyping	*pfhrp*3 genotyping	*n*	Prevalence
Positive	Positive	306	96.5%
Positive	Negative	4	1.3%
Negative	Positive	1	0.3%
—	—	2	0.6%
—	Positive	3	0.9%
Positive	—	1	0.3%
Total		317	

Positive represents the presence of the gene; Negative represents the absence of the gene; — represents missing data.

Kericho site had the highest prevalence of single *pfhrp*3 gene deletion results, at 2.2% (2 of 93 samples) followed by Malindi site at 1.9% (1 of 53 samples). However, the prevalence of single deletion of *pfhrp*3 gene across all study sites was significantly low. The one sample that had single *pfhrp*2 deletion was from the Kombewa study site (1 of 117 samples). No sample had double deletion of *pfhrp*2 and *pfhrp*3 across all study sites.

There was no clear trend in *pfhrp*2 or *pfhrp*3 gene deletions across years, as the prevalence of gene deletions was too low (Table 3). However, *pfhrp*3 single gene deletions were highest in 2013 (3 of 76 samples), the only sample with *pfhrp*2 single gene deletion was from 2016. There was no double deletion of both *pfhrp*2 and *pfhrp*3 genes across all years (Table 3). Of 224 post-RDT samples, one had single deletion of *pfhrp*2, and four had single deletion of the *pfhrp*3 gene only. On the other hand, none of the 93 pre-RDT samples had a deletion of either gene (Table 3).

**Table 3:** Temporal trends of *pfhrp*2 and *pfhrp*3 deletions.

Year	Sample size	*pfhrp*2 deletion	*pfhrp*3 deletion
2003–2005	93	0	0
2013	76	0	3 (3.9%)
2014	46	0	0
2015	58	0	1 (1.7%)
2016	39	1 (2.6%)	0
2017	5	0	0

### *Pfhrp*2 and *pfhrp*3 and their flanking genes deletion profiles

A total of 317 *P. falciparum* PCR-positive samples were genotyped to assess deletions in the *pfhrp*2 and *pfhrp*3 genes and their respective flanking regions. Most samples, 265 (83.6%), had an intact *pfhrp*2 gene, with both the 5′ (Pf3D7_0831900) and 3′ (Pf3D7_0831700) flanking genes present. Single deletions of the 5′ flanking region were observed in 41 samples (12.9%). Two samples had single deletion of the 3′ flanking gene, and 2 samples showed deletions in both the 5′ and 3′ flanking genes, while retaining an intact *pfhrp*2 gene. No sample showed a double deletion involving *pfhrp*2 and any of its flanking genes, and no triple deletions (loss of *pfhrp*2 plus both flanking genes) were identified (Table 4).

**Table 4:** Deletion patterns of *pfhrp*2 and its flanking genes.

5′ flanking gene (Pf3D7_0831900)	Target *pfhrp*2 (Pf3D7_0831800)	3′ flanking gene (Pf3D7_0831700)	*n*	Prevalence
Positive	Positive	Positive	265	83.6%
Negative	Positive	Positive	41	12.9%
Positive	Positive	Negative	2	0.6%
Negative	Positive	Negative	2	0.6%
Positive	Negative	Positive	1	0.3%
Missing data			6	1.9%
Total			317	

A similar pattern was observed for the *pfhrp*3 locus. Most samples 280 (88.3%) showed intact *pfhrp*3 together with both flanking genes (Pf3D7_1372100 and Pf3D7_1372400). Upstream flanking gene deletions, with the *pfhrp*3 gene retained, occurred in 10 samples (3.2%), while downstream flanking gene deletions were detected in 16 samples (5.0%). One (0.3%) sample had double deletion of *pfhrp*3 and the downstream flanking gene, whereas 2 (0.6%) samples had deletion of both upstream and downstream genes with an intact *pfhrp*3 gene. No sample exhibited a triple deletion of *pfhrp*3 and all its flanking genes (Table 5).

**Table 5:** Deletion patterns of *pfhrp*3 and its flanking genes.

5′ flanking gene Pf3D7_1372100	Target *pfhrp*3 (Pf3D7_1372200)	3′ flanking gene (Pf3D7_1372400)	*n*	Prevalence
Positive	Positive	Positive	280	88.3%
Positive	Positive	Negative	16	5.0%
Negative	Positive	Positive	10	3.2%
Positive	Negative	Positive	3	1.0%
Negative	Positive	Negative	2	0.6%
Positive	Negative	Negative	1	0.3%
Missing data			5	1.6%
Total			317	

### Logistic regression analysis of RDT reactivity with respect to *pfhrp*2 and *pfhrp*3 gene deletion status

Samples with parasite densities of at least 30 per microliter by *P. falciparum* qPCR and positive by pan-based RDT result were included in the analysis to assess the relationship between RDT reactivity and *pfhrp*2/*pfhrp*3 deletions (*n* = 195). Among the 21 samples that were negative on the PfHRP2-based RDT, 20 (95.2%) were positive for both *pfhrp*2 and *pfhrp*3 gene, one (4.8%) sample was negative for *pfhrp*3 gene only and none of the 21 samples lacked *pfhrp*2 gene (Table 6).

**Table 6:** *Pfhrp*2 and *pfhrp*3 deletion patterns in pan-RDT positive and PfHRP2-based RDT negative samples.

*pfhrp*2 (Pf3D7_0831800)	*pfhrp*3 (Pf3D7_1372200)	PfHRP2-based RDT positive	Prevalence	PfHRP2-based RDT negative	Prevalence
Positive	Positive	169	97.1%	20	95.2%
Positive	Negative	3	1.7%	1	4.8%
Negative	Positive	1	0.6%	0	0%
Negative	Negative	0	0%	0	0%
Positive	—	1	0.6%	0	0%
Total		174			

— Represents missing data.

None of the false-negative PfHRP2-based RDT samples had a *pfhrp*2 gene deletion. In contrast, *pfhrp*2 deletion was detected in 0.6% (1/173) of samples that were positive by the PfHRP2-based RDT. A single *pfhrp*3 gene deletion with an intact *pfhrp*2 gene was identified in 4.8% (1/21) of the false-negative PfHRP2-based RDT samples and 1.7% of PfHRP2-based RDT positive samples. There was no significant association between false-negative *Pf*HRP2 based RDT results with deletion of either *pfhrp*2 gene (Firth-corrected logistic regression coefficient = 1.02, *P*. value = 0.57) nor *pfhrp*3 (coefficient = 1.6, *P*. value = 0.17) gene (Supplementary Equation S1).

## Discussion

The WHO recommends prompt malaria diagnosis using either microscopy or malaria Rapid Diagnostic Tests (RDTs) before treatment is initiated. Although malaria RDTs were adopted globally for malaria case management in the 2010, Kenya implemented a nationwide roll-out to public health facilities in 2012 (27). This transition improved diagnostic access, with RDTs now accounting for over 60%–80% of all diagnoses. The expanded use of RDTs has strengthened malaria case management by enabling the rapid detection of infections (2). However, the diagnostic accuracy of the common RDTs, those targeting the PfHRP2, can be compromised when parasites carry deletions in the *pfhrp*2 gene. Such deletions and those affecting the related *pfhrp*3 gene, may lead to false-negative test results. Given Kenya's heavy reliance on PfHRP2-based RDTs, continuous monitoring *pfhrp*2 and *pfhrp*3 deletions is essential to ensure the continued reliability of diagnostic testing. We evaluated the prevalence of *pfhrp*2 and *pfhrp*3 deletions in 350 archived samples collected before the national roll-out of RDTs (2003–2005) and in the years following widespread RDT implementation (2013–2017). This approach allowed us to assess whether *pfhrp*2/*pfhrp*3 deletions were present prior to routine RDT use and to determine whether their prevalence has shifted in the post-RDT era.

The prevalence of *pfhrp*2 gene deletion 0.3%, a finding similar to previous studies done in Kenya which have recorded *pfhrp*2 gene deletion prevalence of around 0.0%–6.0% (15–17, 28). On the other hand, the prevalence of *pfhrp*3 gene deletion was 1.3% which is in agreement with previous studies which have recorded a prevalence of 0.7%–2.0% (15, 17). Generally, studies have reported a relatively low prevalence of *pfhrp*2/*pfhrp*3 gene deletion in East Africa, including Kenya but a substantially higher prevalence in parts of the Horn of Africa, including Eritrea with 80% (40/50) false negative RDT result and Djibouti 86.5% (64/74) double deletion of *pfhrp*2/*pfhrp*3 (4, 10, 29).

The low prevalence of *pfhrp*2/*pfhrp*3 gene deletions observed in Kenya likely reflects high malaria transmission intensity, which sustains parasite genetic diversity, frequent polyclonal infections that can mask deleted mutant and limit their spread and reduced selective pressure from PfHRP2-based RDT use. In contrast, the high prevalence reported in the Horn of Africa may be attributed to strong selection pressure driven by prolonged exclusive use of PfHRP2-based diagnostics, coupled with low transmission settings that facilitate clonal expansion of deletion-bearing parasites (10, 29, 30).

Kombewa was the only site with single *pfhrp*2 gene deletion. Kericho site had the highest prevalence of single *pfhrp*3 gene deletion (2.2%) followed by Malindi with 1.9% and Kombewa had the lowest prevalence with 0.9% *pfhrp*3 gene deletion. However, interpretation of these deletions should be done with caution since *pfhrp*3 deletion prevalence was very low across all sites. Nevertheless, it is clear that Kombewa (0.9%) produced the lowest prevalence of *pfhrp*3 gene deletion, despite it having a larger sample size than Kericho and Malindi. These findings demonstrate low *pfhrp*2 and *pfhrp*3 gene deletions during the study period, consistent with low selection pressure on these antigens.

Interestingly, several deletion events involving the upstream and downstream flanking regions of *pfhrp*2 and *pfhrp*3 were identified. For *pfhrp*2, 41 samples had deletion of the 5′ flanking gene, while 4 samples lacked the 3′ flanking gene. The single sample with a *pfhrp*2 gene deletion retained both flanking genes. A similar pattern was observed for *pfhrp*3, 75% (3/4) of samples with a *pfhrp*3 gene deletion had intact 5′ and 3′ flanking regions. However, one sample showed deletion of *pfhrp*3 and its downstream flanking gene, 17 samples showed deletion of the 3′ flanking gene, and 2 samples demonstrated deletion of both the 5′ and 3′ flanking genes while retaining *pfhrp*3. These findings are consistent with flanking-gene deletions occurring independently of causal *pfhrp*2 and *pfhrp*3 gene loss, which may indicate distinct genetic events rather than coordinated multi-gene deletion mechanisms. This pattern contrasts with observations from South America (3, 22, 31, 32). For example, Abdallah et al. (22) reported that among 68 samples collected in Honduras (2008–2009), 26% showed simultaneous deletion of *pfhrp*3 and its 5′ flanking gene (*Pf*3*D*7*_*1372400), and 15% exhibited deletions involving *pfhrp*3 and both flanking genes (22). Similarly, Dorado et al. found that *pfhrp*2-deleted isolates retained the 3′ flanking gene, while 90% of *pfhrp*3-deleted isolates lacked the 5′ flanking region (*Pf*3*D*7*_*1372100) (32). In Peru, Gamboa et al. reported that eight of nine *pfhrp*2-deleted samples lacked the 5′ flanking gene but retained the 3′ region, while *pfhrp*3-deleted isolates showed loss of the 5′ flanking gene, with three additionally lacking the 3′ flanking region (3).

Regarding diagnostic performance, PfHRP2-based RDTs exhibited a sensitivity of 89.2% (174/195) relative to pan-positive detection among *P. falciparum* PCR-confirmed samples with at least 30 parasites/µL. WHO recommended minimum sensitivity of 95% for samples with parasite densities ≥100 parasites/µL (33, 34). Importantly, most of the false-negative samples carried intact *pfhrp*2 and *pfhrp*3 genes, with only 4.8% showing *pfhrp*3 only deletions while retaining *pfhrp*2.

These findings align with observations by Li et al., who reported that samples positive by microscopy and PCR, but negative on two different PfHRP2-based RDTs, possessed intact *pfhrp*2 genes, suggesting causes other than the gene deletions (35).

Interestingly, the *pfhrp*2 only gene deleted sample identified in this study occurred in an RDT-positive sample rather than in a false-negative case. This pattern was also observed in other studies, suggesting that PfHRP2-based RDT positivity can be maintained through cross-reactivity with the paralogous PfHRP3 antigen, particularly when *pfhrp*3 remains intact (3, 4, 7, 10). In this instance, the RDT-positive sample with a *pfhrp*2 deletion had an intact *pfhrp*3 gene, supporting the likelihood that the diagnostic signal was driven by PfHRP3 expression. Another possible explanation is the presence of mixed infections, in which parasites lacking *pfhrp*2 and some with an intact *pfhrp*2 gene; produce PfHRP2 antigen that is sufficient to generate a positive RDT result despite the presence of *pfhrp*2-deleted strains (3, 10, 36).

Temporal analysis of false-negative RDT results from 2013 to 2016 in Kombewa and Kericho did not reveal a consistent trend. In Kombewa, the prevalence of false-negative results increased between 2013 and 2015 before declining in 2016, whereas in Kericho, a steady decrease in false-negative rates was observed over the same period. These fluctuations may partially reflect differences in parasite densities across sites: most false-negative samples from Kericho had slightly lower parasitemia compared with those from Kombewa, which is known to reduce RDT sensitivity even when *pfhrp*2 and *pfhrp*3 are intact. However, the low numbers of false negative RDT samples makes it difficult to draw a conclusion about this finding.

Samples collected between 2003 and 2005 lacked RDT data, as they were collected before the nationwide introduction of RDTs. This limited our ability to compare RDT performance between pre- and post-RDT rollout periods directly. Nonetheless, genotyping of *pfhrp*2 and *pfhrp*3 provided a useful proxy for assessing the potential effects of RDT implementation on gene deletions. Secondly, post-RDT samples (2013–2017) were recruited mainly based on pan/*pfhrp*2-based RDT results, which could have introduced selection bias and overestimation of diagnostic sensitivity or underestimation of gene deletion prevalence, as most samples had high parasitemia and were unlikely to be missed by RDTs. However, because the parent study included all symptomatic individuals, even those with negative pan/Pf bands, some symptomatic malaria cases missed by RDTs were still captured. Third, archived samples were used for RDT reactivity testing, but data on the long-term stability of these samples and the reliability of malaria RDTs on archived specimens are limited. Additionally, differences in malaria transmission intensity across sites and variations in the timing of site enrollment into the original study resulted in uneven sample distribution, constraining spatial and temporal analyses. In polyclonal infections, nested PCR is likely to amplify dominant strains and miss the minor parasites carrying the deletion. Consequently, low-frequency deletion-bearing clones may not be detected, which could lead to an underestimation of deletion prevalence.

## Conclusion

*pfhrp*2 and *pfhrp*3 deletions were extremely rare in the samples analysed, and the patterns observed in their flanking genes suggest independent gene-loss events rather than coordinated multigene deletion mechanisms. Importantly, none of the detected deletions were associated with false-negative PfHRP2-based RDT results. Based on the samples collected after the nationwide rollout, PfHRP2-based RDTs were reliable for malaria diagnosis in the surveyed regions. However, continued molecular surveillance is essential to detect emerging deletion trends early and safeguard the long-term effectiveness of PfHRP2-based RDTs.

## Data Availability

The original contributions presented in the study are included in the article/Supplementary Material, further inquiries can be directed to the corresponding author/s.
