# Supplementary material for: Low prevalence of Plasmodium falciparum pfhrp2 and pfhrp3 gene deletions in archived samples collected before and after nationwide malaria-RDT rollout in Kenya
Source: Front Epidemiol. 2026 Sep 18;6:1817539. doi: 10.3389/fepid.2026.1817539 (PMC13630768; doi:10.3389/fepid.2026.1817539)
Supplement: Supplementary file 1 [file Supplementaryfile1.docx]

**Supplementary Material**

6598 bp

9832 bp

*Pfhrp*2

Histidine rich protein 2

*Pf*3D7_0831800

(1064bp)

HSP70x

Heat shock protein 70

*Pf*3D7_0831700

(2040)

Protein coding pseudogene

*Pf*3D7_0831900

(1002bp)

Supplementary Figure 1: Adapted from Plasmodb release 46 (<http://plasmodb.org/plasmo/>).

Plasmodium exported protein (PHISTb), unknown function *Pf*3D7_1372100

(2006 bp)

5040 bp

2645 bp

4353 bp

*Pfhrp*3

Histidine rich protein 3

*Pf*3D7_1372200

(977 bp)

ACS4

Acyl-CoA synthetase

*Pf*3D7_1372400

(2592 bp)

ACS4

Acyl-CoA synthetase

*Pf*3D7_1372300

(2592 bp)

Supplementary Figure 2: Adapted from Plasmodb release 46 (<http://plasmodb.org/plasmo/>).

**Supplementary Figure 3:** A represents *Pfhrp*2 gene (228bp), B represents *Pfhrp*2, 5’ flanking gene (301bp) while C represent *Pfhrp*2, 3’ flanking gene (198bp).

**C: *Pf*3D7_1372400**

**Supplementary Figure 4:** A represent *Pfhrp*3 gene (225 bp), B *Pfhrp*3 5’ flanking gene (241 bp) and C *Pfhrp*3 3’ flanking gene (225 bp) PCR Result

```
logistf(formula = PfHRP2_RDT ~ Pfhrp2 + Pfhrp3, data = rdt_hrp2_3)

Model fitted by Penalized ML
Coefficients:
                  coef se(coef) lower 0.95 upper 0.95      Chisq
(Intercept) -0.5088725 1.946273 -4.0571405   4.767348 0.06512197
Pfhrp2       1.0196981 1.649640 -3.9768032   3.984569 0.31873693
Pfhrp3       1.6074848 1.058920 -0.8183788   3.674030 1.91026929
                    p method
(Intercept) 0.7985763      2
Pfhrp2      0.5723677      2
Pfhrp3      0.1669333      2

Method: 1-Wald, 2-Profile penalized log-likelihood, 3-None

Likelihood ratio test=2.198206 on 2 df, p=0.3331698, n=194
Wald test = 82.81258 on 2 df, p = 0
```

**Supplementary equation 1:** A summary of Firth-corrected logistic regression *Pf*HRP2 based RDT result against the *Pfhrp*2 and *Pfhrp*3 gene genotyping
